# Presence of lacunar infarctions is associated with the spatial navigation impairment in patients with mild cognitive impairment: a DTI study

**DOI:** 10.18632/oncotarget.13409

**Published:** 2016-11-16

**Authors:** Yan-Feng Wu, Wen-Bo Wu, Qing-Ping Liu, Wen-Wen He, Hong Ding, Zuzana Nedelska, Jakub Hort, Bing Zhang, Yun Xu

**Affiliations:** ^1^ Department of Neurology, the Affiliated Drum Tower Hospital of Nanjing Medical University, Nanjing, Jiangsu, P. R. China; ^2^ Department of Neurology, the Second Affiliated Hospital of Nanjing Medical University, Nanjing, Jiangsu, P. R. China; ^3^ Department of Radiology, The Second Affiliated Hospital of Nanjing Medical University, Nanjing, Jiangsu, P. R. China; ^4^ Department of Neurology, Memory Disorders Clinic, Charles University in Prague, 2nd Faculty of Medicine and Motol University Hospital, Prague, Czech Republic; ^5^ International Clinical Research Center, St.Anne's University Hospital Brno, Brno, Czech Republic; ^6^ Department of Radiology, The Affiliated Drum Tower Hospital of Nanjing University Medical School, Nanjing, Jiangsu, P. R. China; ^7^ Jiangsu Key Laboratory for Molecular Medicine, Nanjing University Medical School, Nanjing, China; ^8^ Nanjing Medical Research Center on Neurology and Psychiatry, Jiangsu, P. R. China

**Keywords:** lacunar infarction, mild cognitive impairment, spatial navigation, diffusion tensor imaging, Gerotarget

## Abstract

Lacunar cerebral infarction (LI) is one of risk factors of vascular dementia and correlates with progression of cognitive impairment including the executive functions. However, little is known on spatial navigation impairment and its underlying microstructural alteration of white matter in patients with LI and with or without mild cognitive impairment (MCI). Our aim was to investigate whether the spatial navigation impairment correlated with the white matter integrity in LI patients with MCI (LI-MCI). Thirty patients with LI were included in the study and were divided into LI-MCI (*n*=17) and non MCI (LI-Non MCI) groups (*n*=13) according neuropsychological tests.The microstructural integrity of white matter was assessed by calculating a fractional anisotropy (FA) and mean diffusivity (MD) from diffusion tensor imaging (DTI) scans. The spatial navigation accuracy, separately evaluated as egocentric and allocentric, was assessed by a computerized human analogue of the Morris Water Maze tests Amunet. LI-MCI performed worse than the CN and LI-NonMCI groups on egocentric and delayed spatial navigation subtests. LI-MCI patients have spatial navigation deficits. The microstructural abnormalities in diffuse brain regions, including hippocampus, uncinate fasciculus and other brain regions may contribute to the spatial navigation impairment in LI-MCI patients at follow-up.

## INTRODUCTION

Lacunar infarcts (LI) are small subcortical infarcts resulting fromocclusionof one of the penetrating arteries, which comprise approximately % of allischemic strokes [1]. The symptomatic LI have a favorable short-term prognosis, but the mid- and long-term prognosis is poor because of recurrence of vascular cognitive impairment, stroke and increased risk of death [1]. The multinational Leukoaraiosis and Disability study showed that an increase in silent lacunes parallels significantly with cognitive decline, especially in executive functions [2]. Symptomatic LI particularly predispose to vascular dementia [3]. It should be noted that most LI are asymptomatic, and discovered by Magnetic Resonance Imaging (MRI), accidentally, in 20-28% of the population of over 65 years of old. Some studied have identified silent lacunar infarction as a risk factor for cognitive impairment [2, 4]. Therefore, the identification of appropriate neuropsychological and neuroimaging markers to detect early cognitive decline in patients with lacunar infarction and track the disease progression is crucial.

The cognitive consequences of LI are more related to subcortical function, i.e. affecting executive functions and information processing speed, such as inhibition set-shifting, selective attention and flexibility [1, 2]. Spatial navigation is a complex cognitive skill which is potentially specific to cortical structures, necessary for everyday functioning and includes several cognitive abilities such as executive planning, attention, spatial memory, route learning, visual imagery, scene recognition and orientation [5]. We can distinguish egocentric (route or body centered) and allocentric (world centered) spatial navigation strategy [6, 7]. Egocentric navigation is body centered and localized mostly in parietal cortex and striatum, while the allocentric navigation is world centered and it is dependent mostly on the hippocampus [6, 7] Impaired spatial navigation is a frequently reported symptom of MCI and Alzheimer's disease (AD) patients in both real-space and virtual environments, with similar results [8]. For example, Czech study [9] used a human analog of the Morris Water Maze (hMWM) to demonstrate a spatial navigation impairment in patients with multiple-domain amnestic mild cognitive impairment (aMCI) of a similar magnitude to that found in AD patients. Follow-up studies showed that spatial navigation testing using hMWM can identify amnestic MCI (aMCI) patients at high risk of conversion to dementia, specifically aMCI patients with a hippocampal type of memory impairment, who were similarly impaired in hMWM spatial test than patients with mild AD [10, 11]. Besides the human analog of the MWM, the computerized hMWM has also the potential to measure the effects of donepezil in mild AD and could be used as a sensitive cognitive outcome measure in AD clinical trials [12]. However, to the best of our knowledge, none of spatial navigation study was employed in patients with lacunar infarction. Little is known about the spatial navigation performance in patients with the vascular or the complex mixture of vascular and AD neuropathology.

The study of structure basis of spatial navigation indicated that egocentric navigation is localized mainly in parietal cortex and striatum, while the allocentric navigation dependents mainly on the hippocampus [6, 7]. However, little is known about the microstructural integrity of white matter and possible spatial navigation impairment. Diffusion tensor imaging (DTI) is usually used to assess the microstructural integrity of the whole brain white matter. The most commonly used DTI metrics are fractional anisotropy (FA), which measure the directionality of water diffusion, and mean diffusivity (MD), a measure of averaged the magnitude of diffusion in all spatial directions. Elevated MD and decreased FA are thought to reflect progressive loss of the barriers restricting the motion of water molecules in tissue compartments associated with the neuronal loss and disruption of myelin sheaths in dementia [13].

Although a number of previous studies have indicated correlations between cognition (particularly executive function) and both MD and FA in cerebral small vessel diseases [14, 15], there are few studies concerning the influence of brain microstructural abnormality on spatial navigation in patients with LI. Therefore, our purpose was to investigate whether the spatial navigation impairment would be correlated with the structural integrity of white matter in patients with LI and concomitant MCI (LI-MCI). The first aim of this study was to characterize the profile of spatial navigation performance in LI patients with or without MCI. We hypothesized that LI patients with MCI (LI-MCI) and also those without MCI (LI-NonMCI) would perform worse on spatial navigation tests compared with cognitively normal controls (CN). The second aim was to evaluate the patterns of microstructural abnormality of brain white matter as detected using DTI in LI patients. The third aim was to assess whether the baseline brain microstructural abnormality would be associated with the performance on the allocentric and egocentric spatial navigation tests at the follow-up.

## RERULTS

### Demographic and neuropsychological tests

In the present study, 17 patients with LI (56.67%) fulfilled the criteria of MCI. No significant differences were found in gender, age, education level, smoking and drinking habit, diabetes mellitus history, but the history of hypertension (*t* = 9.24, *P* = 0.004) among groups (Table 1). MMSE and MoCA scores were both significantly lower in the patient group *versus* CN. Patients showed no deficits in the other neuropsychological tests. When the patients with LI were divided into LI-MCI and LI-Non MCI groups, those with concomitant MCI had also significantly lower scores on MMSE and MoCA compared with LI-NonMCI and CN group. Furthermore, patients with LI-MCI performed worse than LI-NonMCI and CN on Symbol digit modalities test (F = 4.76, *P* = 0.01), Reversed digit span total numbers recalled (F = 3.67, P = 0.03), Verbal fluency test (F = 6.61, *P* < 0.01), AVLT delayed recall (F = 3.54, *P* = 0.04) and recognition (F = 9.23, *P* < 0.01) (Table 1).

**Table 1:** Demographic and test data for cognitively normal controls and all patients with lacunar infarction

Characteristics	CN	LI	LI-Non MCI	LI-MCI	t/c2	P1	F/c2	P2
	(*n*= 14)	(*n*= 30)	(*n*= 13)	(*n*= 17)				
Gender(male/female)	6/8	19/11	9/4	10/7	1.63	0.33	1.96	0.38
Age(years)	60.64±7.81	62.40±8.67	64.15±7.79	61.06±9.23	0.65	0.52	0.71	0.50
Education(years)	11.21±3.87	9.73±2.86	10.85±2.67	8.88±2.78	1.43	0.16	2.51	0.09
Hypertension	6	26	10	16	9.24	0.004	10.35	0.01
Diabetes Mellitus	3	12	4	8	1.47	0.31	2.34	0.31
Daily Smoker	6	12	4	8	0.01	0.93	1.10	0.58
Drinking	5	10	3	7	0.02	0.88	1.10	0.58
Systolic BP(mmHg)	126.78±10.48	136.17±10.47	135.00±11.55	137.06±9.85	1.89	0.08	2.56	0.09
Diastolic BP(mmHg)	80.71±9.97	82.33±8.28	81.92±9.02	62.65±7.93	0.57	0.57	0.18	0.84
LDL(mmol/L)	2.49±0.69	2.63±0.67	2.49±1.17	2.74±0.66	0.61	0.55	0.68	0.51
FBS(mmol/L)	5.45±0.80	5.95±1.57	5.56±1.17	6.25±1.79	1.12	0.27	1.61	0.21
Lacune Count		2.63±1.52	2.69±1.60	2.58±1.50	0.03	0.86		
Lacune location					4.53	0.10		
Anterior		22	12	10				
Thalamic		3	0	3				
Posterior		5	1	4				
MMSE	28.93±0.92	27.70±2.00	28.31±1.70	27.24±2.14	2.79	0.01	3.95	0.03
MoCA	25.64±1.55	24.10+3.18	27.00±1.29	21.88±2.23	2.17	0.04	33.75	<0.01
CDT	4.0±0	3.67±0.90	4.00±0	3.06±1.03	3.25	0.003	11.18	<0.01
Symbol digit modalities test	33.64±6.61	29.9±11.72	35.62±11.57	25.53±10.09	1.11	0.27	4.76	0.01
Digit span total numbers recalled	6.50±1.40	6.43±1.43	7.07±1.44	5.94±1.25	0.15	0.89	2.60	0.09
Reversed digit span total numbers recalled	4.14±0.95	3.97±0.96	4.46±0.52	3.59±1.06	0.57	0.57	3.67	0.03
Verbal fluency test	27.71±5.95	24.80±6.17	28.54±5.85	21.94±4.83	1.48	0.15	6.61	<0.01
RAVLT								
Immediate recall	5.83±1.32	5.62±1.63	6.20±1.12	5.18±1.84	0.42	0.68	1.82	0.18
Delayed recall (after 5min)	6.64±1.50	5.27±2.89	5.85±2.82	4.82±2.94	1.67	0.10	2.01	0.15
Delayed recall (after 20min)	6.79±2.36	5.57±3.55	7.08±3.50	4.41±3.22	1.17	0.25	3.54	0.04
Word recognition	22.93±1.59	22.27±1.68	23.46±1.05	21.35±1.50	1.24	0.22	9.23	<0.01
TMT-A (sec)	75.43±20.39	87.21±40.23	83.08±33.99	90.37±45.19	1.03	0.31	0.68	0.51
TMT-B (sec)	145.36±43.60	171.06±53.70	144.31±32.71	191.51±58.29	1.56	0.13	5.07	0.01
HAMD-17	1.14±1.10	1.10±1.53	0.92±1.80	1.18±1.33	0.17	0.87	0.13	0.88

Abbreviation: CN, cognitively normal controls; LI, lacunar infarction ; LI-Non MCI, lacunar infarction patient with non mild cognitive impairment; LI-MCI, lacunar infarction patient with mild cognitive impairment; LDL, low-density lipoprotein; FBS, fasting blood sugar; MMSE, mini-mental state examination; MoCA, Montreal Cognitive Assessment; CDT, Clock Drawing Task; RAVLT, Rey Auditory Verbal Learning Test; TMT-A, Trail Making Test A; TMT-B, Trail Making Test B; HAMD-17, 17-item Hamilton Depression Rating Scale. *P_1_*, patients group compared with control group; *P_2_*, patients with MCI compared with control group and patients without MCI.

### Comparison of the fractional anisotropy and mean diffusivity values between cognitively normal controls and all patients with lacunar infarction

Mean FA values in the LI-MCI group were lower than the LI-Non MCI and CN groups across several brain regions including the left retrolenticular, left posterior thalamic radiation, left sagittal stratum, right external capsule, left cingulated gyrus, left hippocampus, left inferior fronto-occipital fasciculus, right inferior fronto-occipital fasciculus, left uncinate fasciculus, right uncinate fasciculus, right anterior thalamic radiation, right corticospinal tract, left inferior longitudinal fasciculus (Table 2). Mean MD values were higher in the LI-NonMCI group in the same regions as FA compared with other two groups, and statistically significant associations were found in the right inferior fronto-occipital fasciculus (F = 3.98, *P* = 0.03) and right uncinate fasciculus (F = 4.56, *P* = 0.02).

**Table 2:** Comparison of the fractional anisotropy and mean diffusivity values between cognitive normal controls and all patients with lacunar infarction

	CN	LI		F	*P*
	(*n*= 14)	LI-No (*n*= 13)	LI-MCI (*n*= 17)		
FA					
Retrolenticular part of internal capsule, left	0.48±0.04	0.50±0.04	0.46±0.05	3.93	0.03
Posterior thalamic radiation, left	0.46±0.03	0.47±0.04	0.43±0.04	4.21	0.02
Sagittal stratum, left	0.42±0.03	0.44±0.04	0.39±0.05	5.50	0.01
External capsule, right	0.33±0.03	0.34±0.03	0.30±0.04	4.11	0.02
Cingulum (cingulated gyrus), left	0.31±0.02	0.33±0.04	0.29±0.04	3.53	0.04
Cingulum (hippocampus), left	0.27±0.03	0.27±0.05	0.24±0.04	3.44	0.04
Inferior fronto-occipital fasciculus, right	0.39±0.03	0.41±0.04	0.36±0.05	4.95	0.01
Inferior fronto-occipital fasciculus, left	0.37±0.04	0.39±0.04	0.35±0.04	3.70	0.03
Uncinate fasciculus, right	0.41±0.03	0.44±0.05	0.39±0.06	4.42	0.02
Anterior thalamic radiation, right	0.33±0.03	0.33±0.04	0.29±0.04	3.40	0.04
Corticospinal tract, right	0.50±0.03	0.52±0.04	0.47±0.05	3.83	0.03
Inferior fronto-occipital fasciculus, left	0.38±0.02	0.39±0.03	0.35±0.04	4.96	0.01
Inferior longitudinal fasciculus, left	0.38±0.03	0.39±0.03	0.36±0.04	4.18	0.02
Uncinate fasciculus, left	0.35±0.03	0.37±0.03	0.33±0.04	3.86	0.03
Uncinate fasciculus, right	0.35±0.03	0.37±0.04	0.33±0.04	6.05	0.01
MD					
inferior fronto-occipital fasciculus, right	0.89*10-3±0.06*10-3	0.87*10-3±0.06*10-3	0.94*10-3±0.09*10-3	3.98	0.03
uncinate fasciculus, right	0.87*10-3±0.05*10-3	0.87*10-3±0.06*10-3	0.97*10-3±0.16*10-3	4.56	0.02

Abbreviation: CN, cognitive normal controls; LI, lacunar infarction; LI-No, lacunar infarction non-mild cognitive impairment; MCI, mild cognitive impairment; FA, fractional anisotropy; MD, mean diffusivity.

### Spatial navigation impairment

Independent t-test analysis was used to evaluate differences between patient and control groups in average total errors on the spatial navigation tasks (Table 3). The patient group (LI-MCI and LI-nonMCI combined) performed worse on all spatial tests: the mixed allocentric-egocentric, egocentric, allocentric and delayed subtest (represented by an average total error across 8 trials within a respective test) compared to CN group, but these differences were not significant (*P* > 0.05) while only delayed subtest showed difference (*t* = 2.81, *P* = 0.01). Further, ANOVA and post-hoc contrast analyses were used to examine spatial navigation performance in the LI-MCI and LI-NonMCI groups compared to CN. There were significant main effects for group in egocentric (F = 3.46, *P* = 0.04), and delayed recall (F = 5.62, *P* = 0.01) spatial navigation subtests. The LI-MCI group performed worse than the CN and LI-NonMCI groups on egocentric and delayed recall spatial navigation subtests.

**Table 3:** Spatial navigation tests for cognitively normal controls and lacunar infarction patients

Characteristics	CN	LI-Non MCI	LI-MCI	F	*P*
	(*n*= 14)	(*n*= 13)	(*n*= 17)		
Average total error					
AEV	34.22±15.67	33.80±18.56	45.51±27.59	1.45	0.25
EV	49.41±37.71	45.67±29.59	80.85±49.97	3.46	0.04
AV	41.09±21.63	36.65±18.46	64.01±53.91	2.42	0.10
DV	22.93±8.44	27.75±15.04	64.22±58.47	5.62	0.01
Average duration					
AEV	10.00±3.10	10.02±2.40	8.47±2.65	1.65	0.21
EV	9.84±4.25	10.59±5.09	10.57±7.51	0.08	0.93
AV	10.40±3.77	13.13±4.77	10.13±6.49	1.39	0.26
DV	7.68±3.30	9.93±4.01	16.18±36.16	0.59	0.56
Average angular deviation					
AEV	7.44±4.90	7.06±5.01	10.68±7.63	1.64	0.21
EV	13.38±11.84	8.58±4.90	31.26±54.69	1.81	0.18
AV	8.26±5.11	7.17±4.56	27.8±63.83	1.32	0.28
Average distance deviation					
AEV	21.96±10.50	13.73±7.67	20.53±9.37	3.03	0.06
EV	22.10±11.36	17.91±6.32	21.81±10.38	0.79	0.46
AV	23.63±12.74	19.54±8.61	26.89±16.67	1.08	0.35

Abbreviation: CN, cognitively normal controls; LI-Non MCI, lacunar infarction patient with non mild cognitive impairment; LI-MCI, lacunar infarction patient with mild cognitive impairmen AVE, allo-ego vision; EV, ego vision; AV, allo vision; DV, delayed vision.

### Relationship between DTI metrics and spatial navigation impairment in patients with lacunar infarction and MCI

The relationship between DTI metrics in atlas-based brain regions and egocetric and allocetric scores (represented by an average total error) were examined with linear mixed models (Table 4 and Table 5). Lower FA value indicating abnormal white-matter integrity in the left inferior fronto-occipital fasciculus (IFOF), left uncinate fasciculus and higher MD scores, indicating more microstructural abnormalities, in the bilateral anterior thalamic radiation (ATR) were predictive of higher egocentric navigation scores. Similarly, lower FA value in the right posterior limb of internal capsule, right retrolenticular part of internal capsule, bilateral corticospinal tracts, bilateral hippocampus and higher MD scores in the bilateral ATR were predictive of worse allocentric navigation performance.

**Table 4:** Regression coefficients (linear regression analysis) between egocentric in patients and DTI markers

DTI markers	egocentric	
	Model1	Model2
	Beta (*p* value)	Beta (*p* value)
FA Inferior.fronto-occipital.fasciculus, left	0.23(0.07)	0.22(0.04)
FA Cingulum.(hippocampus),left	0.30(0.03)	0.30(0.03)
FA Uncinate.fasciculus, left	0.26(0.04)	0.29(0.02)
MD Anterior.thalamic.radiation, left	0.34(0.01)	0.31(0.02)
MD Anterior.thalamic.radiation, right	0.27(0.04)	0.23(0.07)

Mode2 has been adjusted by gender, age, education, the history of hypertension, diabetes mellitus, daily Smoking and drinking.

**Table 5:** Regression coefficients (linear regression analysis) between allocentric in patients and DTI markers

DTI markers	allocentric	
	Model1	Model2
	Beta (*p* value)	Beta (*p* value)
FA Posterior limb of internal capsule, right	0.27(0.04)	0.35(0.01)
FA Retrolenticular part of internal capsule, right	0.31(0.02)	0.42(0.01)
FA Corticospinal tract, left	0.30(0.02)	0.37(0.01)
FA Corticospinal tract, right	0.28(0.03)	0.34(0.01)
FA Cingulum (hippocampus), left	0.27(0.04)	0.28(0.04)
FA Cingulum (hippocampus), right	0.33(0.01)	0.37 (0.01)
MD Anterior thalamic radiation, left	0.34(0.01)	0.31(0.02)
MD Anterior thalamic radiation, right	0.31(0.02)	0.26(0.05)

Mode2 has been adjusted by gender, age, education, the history of hypertension, diabetes mellitus, daily Smoking and drinking.

## DISCUSSION

This study explored the effects of brain microstructural alterations on spatial navigation and cognitive impairment in patients with LI and without additional MCI. The results indicated that an egocetric spatial navigation was impaired in patients with both LI and MCI compared to patients with LI but without MCI and cognitively normal controls. We also found different distribution of microstructural abnormalities in certain brain regions in patients with both LI and MCI compared with cognitively normal controls. Furthermore, microstructural abnormalities in many regions, including hippocampus and uncinate fasciculus, may contribute to the spatial navigation impairment at mid-term follow-up.

In line with prior studies, we divided the patients with LI group into those with cognitive impaired (represented by MCI) and cognitively intact subgroups based on neuropsychological testing. 56.67% patients with LI fulfilled the criteria of MCI, which was consisted with the previous report [16]. In our sample, patients LI and MCI performed worse on the symbol digit modalities test, reversed digit span total numbers recalled, verbal fluency test, RAVLT delayed recall and recognition test. Previously, patients with brain infarction generally performed worse than controls in various aspects of cognitive function, such as working memory, the temporary storage of complex information and cognitive flexibility [17, 18]. Our findings also indicated a specific deficit in egocentric spatial navigation strategy in patients with LI and MCI compared to patients with LI but without MCI and cognitively normal controls. Several studies have revealed that patients with aMCI and AD have deficit in spatial orientation [9], which was one of the predictors of clinical progression from aMCI to AD [9, 19]. In a study concerning hippocampal aMCI (HaMCI) [10], the results showed that the HaMCI group (which might represent preclinical AD) presented relatively severe spatial navigation impairment, especially in the allocentric task. Furthermore, the HaMCI group was also impaired in the egocentric task which indicated extra-hippocampal impairment. In addition, Amlerova and colleagues also have demonstrated that allocentric navigation impairment in temporal lobe epilepsy patients and indicated that not only temporal lobe dysfunction itself but also low general cognitive abilities may contribute to the navigation impairment [6]. Therefore, studies of spatial navigation may provide a better understanding of the orientation impairment that occurs in normal aging, cognitive impairment due to AD and vascular type MCI and may be one of the predictors of clinical progression from MCI to dementia [10].

Allocentric and egocentric strategies are likely regulated by different neural structures, including hippocampus and caudate nucleus, respectively [20], [21, 22]. Successful navigation in older individual may be associated with distributed fronto-striatal and striato-hippocampal systems [22]. In our study, we found that MCI group exhibited spatial navigation impairment, mainly in the egocentric task and delayed task, which indicated both intra and extra hippocampal impairment. We demonstrated that LI patients have low FA in the left inferior fronto-occipital fasciculus, left hippocampus, left uncinate fasciculus and high MD in the bilateral anterior thalamic radiation that are predictive of greater error on spatial test, indicating that the loss of microstructural integrity in these regions might be involved in the worse egocentric spatial navigation performance. Egocentric navigation has been associated with cortico-striatal brain regions. Wolbers et al. suggested that the inferior parietal cortex participated in the encoding of spatial association between serial landmarks in an egocentric reference frame, which was relative to the observer's direction when facing the first landmark [23]. The present study also found the association between allocentric navigation and the posterior limb of internal capsule, retrolenticular, corticospinal tract, hippocampus and anterior thalamic radiation in LI patients. One of the first studies using DTI has demonstrated that individuals with high FA in the right hippocampus could more quickly establish a cognitive map of the environment and could more capably use this map for allocentric navigation than individuals with low FA [24]. The results of above studies illustrate that allocentric navigation and the ability to computing the best route toward a goal based on a cognitive map of the environment are sustained by the hippocampus [22].

To our knowledge, our study is the first to report the role of IFOF, ATR, uncinate fasciculus and hippocampus in the egocentric navigational deficit and internal capsule, corticospinal tract, hippocampus and anterior thalamic radiation in the allocentric navigational deficit in LI patients. Our findings agree with a number of studies which have proposed that damage to the hippocampus leads to spatial navigation impairment. A previous structural neuroimaging study demonstrated that allocentric and egocentric navigation impairment toward a goal in a familiar virtual environment was relevant to bilateral hippocampal atrophy, the right inferior parietal cortex and the right precuneus in aMCI patients [25]. The decreased volume of right precuneus was one of the predictor of egocentric impairment [25]. Another study showed that worse spatial navigation performance of MCI and AD was associated with the right posterior parietal cortex and right posterior hippocampal atrophy [26]. The volume of these structures was found to correlate with the accuracy of oriented landmarks on a map [22]. The abnormality of IFOF has been reported to be associated with impaired episodic memory and executive function [27] and the uncinate fasciculus has frequently been associated with the retrieval of autobiographical memories [28]. ATR is a major white matter tract projection from the thalamus that penetrates the anterior limb of the internal capsule, carrying reciprocal connections from the hypothalamus and limbic structures to the frontal cortex [29]. The thalamus has been found to be related to lots of cognitive functions such as episodic memory, executive function and so on [27]. Therefore, in the LI patients, microstructural abnormalities in many regions, including hippocampus, inferior fronto-occipital fasciculus, internal capsule, uncinate fasciculus and anterior thalamic radiation may contribute to the navigation impairment. As is well-known that egocentric navigation is associated with parietal cortex and parietal and temporal lobe abnormalities are considered as a hallmark of AD [10]. Previous studies also demonstrated that navigational defects in AD and MCI patients are associated with parietal lobes and/or the junction of the parietal-occipital-temporal cortex [10, 30]. However, structural correlations of allocentric and egocentric navigation in MCI, AD and other dementia individuals are still underexplored. Further studies which focus on more complex structural correlates of allocentric and egocentric navigation, especially in cognitively impaired individuals, may shed light to this complex issue [7].

There are some limitations in the present study. The primary shortcoming of the present study is that the spatial navigation was only assessment at the follow-up time ranged from 6 to 14 months, not in baseline. Nevertheless, it supplies a preliminary assessment and confirmation of the hypothesis that impairment in spatial navigation was influenced by the brain microstructural abnormality. Further prospective longitudinal studies with age-matched healthy controls and standardized neuropsychological scales that are specific for mild cognitive impairment in patient with lacunar infarction would be necessary for us to better evaluate the timing of neuroanatomical changes relative to cognitive impairments and to validate whether the acknowledged biomarkers which could be used for early detection [11]. Second, the computerized test based on the MWM paradigm may be a useful tool for evaluation of spatial navigation deficits [8]. However, it should be noted that the real-space and computerized 2-dimensional versions are not fully interchangeable, as the computerized spatial navigation tasks lack vestibular and proprioceptive feedback that is normally available in the real-world navigation tasks and that contributes to successful navigation [9]. Furthermore, our study only assessed the association between the structural integrity of white matter and spatial navigation in lacunar infarction patients with MCI. Until now, rare studies have explored the associations between structural and functional alterations and navigation impairment in MCI and dementia. It has become necessary to combine the neuroimaging techniques and neurocognitive methods in further studies [22, 31].

Together, these data suggest that LI patients with concomitant MCI have spatial navigation impairment and also emphasized that microstructural abnormality in many regions including hippocampus may contribute to the navigation impairment. Furthermore, navigation task should use to predict the clinical progression from MCI to dementia in a larger longitude sample.

## MATERIALS AND METHODS

### Participants

Forty-four right-handed participants gave informed consent to participate in this study that was approved by the Second Affiliated Hospital of Nanjing Medical University Research Ethics Committee. Thirty patients with at least one single chronic lacunar infarction were recruited consecutively in the study by the Department of Neurology of Second Affiliated Hospital of Nanjing Medical University between January 2013 and May 2014. Presence and location of lacunes were recorded by a consultant neuroradiologist, based on T_1_W, T_2_W, FLAIR images and diffusion weighted imaging (DWI). A chronic lacune was defined as a cerebrospinal fluid-filled cavity, not hyper-intense on DWI, 3 to 15 mm in diameter, with a surrounding rim of high signal intensity on FLAIR following a vascular distribution [32]. Exclusion criteria were an acute infarction with hyper-intense on DWI; intracerebral hemorrhage or cortical and/or subcortical nonlacunar infarcts identified by MRI; severe renal, cardiovascular, neoplastic hepatic, or chronic disease; major psychiatric diseases; major depression, and dementia.

We dichotomized the patients with LI into LI-MCI group (*n* = 17) and LI-Non MCI (*n* = 13) group according to cognitive status. Mild cognitive impairment (MCI) of the vascular type was considered in non-demented patients exhibiting cognitive impairment mainly as a prominent dysexecutive syndrome and clinical and radiological manifestations of subcortical cerebrovascular disease [33, 34]. MCI of the vascular type was identified according to criteria of Frisoni et al. [34]. It briefly described that the scores not more than −1.5 SD according to gender- and age- matched standardized cognitive normals on at least one of the following tests: Trail Making Test A and B (TMT-A, TMT-B), verbal fluency. Patients within this group also scored equal to or less than −1 SD on one of the following declarative memory tests: immediate, delayed recall or recognition of Rey Auditory Verbal Learning Test (RAVLT) [33].

Cognitively normal controls (CN; *n* = 14) denied having any memory problem and lacunar infarction indentified by MRI. All neuropsychological assessment, spatial navigation testing and MRI were performed at least 3 months post-stroke to minimize acute effects of stroke on cognition. These individuals were recruited from relatives of staff and patients and were selected to have a similar age, education, and gender ratio as the patient group (Table 1).

### Neuropsychological assessment

Neuropsychological assessment was administered within 2 weeks after or before MRI by neuropsychologists. The tests included the Mini-Mental State Examination (MMSE), The Montreal Cognitive Assessment (MoCA), Clock Drawing Task (CDT), Symbol digit modalities test, Digit span total numbers recalled, Reversed digit span total numbers recalled, Rey Auditory Verbal Learning Test (RAVLT), Trail Making Test A and B (TMT-A, TMT-B), Phonetic Verbal Fluency Test, and 17-item Hamilton Depression Rating Scale (HAMD-17).

### MRI analysis and DTI processing

MRI imaging was performed on a on a 3T GE Signa Excite HD 12.0 Twin Speed scanner (General Electric, Milwaukee, WI), which included a maximum slew rate of 150T/m/s and maximum gradient amplitude in each orthogonal plane of 50 mT/m (zoom mode) using 8-channel head coil. All scans were prescribed parallel to the subcallosal line in an axial-oblique orientation, including the following sequences: (1) Axial Fluid Attenuated Inversion Recovery (FLAIR) sequence: TR = 2000ms, TE = 7.4ms, matrix = 256х160, FOV = 240mmх240mm, NEX = 1.0, gap = 0, thickness = 5mm. (2) Coronal spoiled gradient recalled echo T1-weighted (SPGR) sequence: TR = 9.9ms, TE = 2.1ms, flip angle = 15, maxtrix = 256×192, FOV = 240 mm×240 mm, thickness = 2.0 mm, NEX = 1.0, gap = 0. (3) DTI sequence: TE/TR = 73/8,200 ms, 96×96 matrix, 32×24 cm FOV (due to pFOV = 0.75), FA = 90^o^; ETL = 1, slice thickness 3 mm with no gap, resulting in an in-plane resolution size of 3.33 mm×3.33 mm, b-value of 1000, 15 directions, and 1 repetition. All participants were scanned on the same scanner.

DTI data were post processed by PANDA (a pipeline toolbox for analyzing brain diffusion images) [35]. The main procedure of PANDA including: (1) Converting DICOM files into NifTI images; (2) Estimating the brain mask by using the bet command of FSL; (3)Cropping the raw images : to cut off non-brain space in the raw images; (4) Correcting for the eddy-current effect by using the FLIRT and the eddy_correct of FSL command; (5) Calculating diffusion tensor (DT) metrics: this step involves a voxel-wise calculation of the tensor matrix and the DT metrics, including FA and MD. The dtifit command of FSL was applied (6) Output for atlas-based analysis: in addition to the popular voxel-based method of analysis, diffusion metric can be analyzed at the level of region of interest (ROI), which may provide better statistical sensitivity. PANDA calculated the regional diffusion metrics (FA and MD) by averaging the values within each region of the ICBM template, which was used in previous study [36].

### Spatial navigation testing at follow-up

The spatial navigation performance was assessed at follow-up ranged from 6 months to 14 months after MRI examination during March to November 2014. All subjects denied having any progressed memory problem and acute lacunar infarction symptoms. Spatial navigation was tested by PC tests Amunet (NeuroScios GmbH, Austria) which are human analogue of the Morris Water Maze (MWM) task. Studies with the same paradigm, but using different software were published previously [6, 8, 37]. The Amunet tests are designed to separate two different strategies of spatial navigation, egocentric that relies on individual's position and allocentric strategy centered on the object rather than on the observer [22]. The main task of the subjects was to locate an invisible goal in four different subtests using start position and/or two distal orientation cues. The first “allocentric + egocentric” subtest involveed locating the goal using its spatial relationship with both start position and the two distal orientation cues. The second “egocentric” subtest involved using only the start position to locate the goal with no distal orientation cues displayed. The following “allocentric” subtest involved using two distal orientation cues at the “arena walls” for navigation with the start position unrelated to the goal position. The last “delayed” subtest involved the same design, as the “allocentric” subtest but is administered 30min after the end of the allocentric subtest. Each subtest consisted of eight trials, except of delayed subtest which consisted of only two trials [8]. The index was averaged across all eight trials from each of the subtest. The distance between the subjects' choice and the correct goal location measured in pixels was used in the analysis as a main index (average total error) for measuring of spatial navigational ability [8]. Other indexes, such as the time, which the subjects cost in each trial, the total time which the subjects cost in each subtest (average duration), path difference (average distance deviation), and angular difference (average angular deviation), were also measured. In the present study, average total error was used in the statistical analysis as a measure of navigational accuracy.

### Statistical analysis

Statistical analysis was performed using Statistical Package for Social Sciences version 13.0 (SPSS, Chicago, IL). Neuropsychological measurements, spatial navigation scores and DTI metrics of FA and MD are reported as the mean ± SD. Demographic variables between patients and controls were compared using t-tests or χ^2^ test as appropriate. A two-factor ANOVA test was used to investigate the differences of psychophysical assessments, spatial navigation scores and DTI metrics among controls, patients with or without cognitive impairment. Linear regression analysis was carried out between egocentric or allocentric navigation scores and DTI metrics in patients with LI and MCI. For all analyses involving cognitive indices, age, and gender were used as covariates. P-values less than 0.05 were regarded as significant.
